# The Key to Unlocking the Chemotherapeutic Potential of PPAR**γ** Ligands: Having the Right Combination

**DOI:** 10.1155/2012/946943

**Published:** 2012-07-02

**Authors:** Graham Skelhorne-Gross, Christopher J. B. Nicol

**Affiliations:** ^1^Department of Pathology and Molecular Medicine, Queen's University, Kingston, ON, Canada K7L 3N6; ^2^Cancer Biology and Genetics Division, Cancer Research Institute, Queen's University, Kingston, ON, Canada K7L 3N6; ^3^Department of Biomedical and Molecular Sciences (Pharmacology and Toxicology), Queen's University, Kingston, ON, Canada K7L 3N6

## Abstract

Despite extensive preclinical evidence that peroxisome proliferator-activated receptor (PPAR)**γ** activation protects against tumourigenesis, results from a few clinical trials using PPAR**γ** ligands as monotherapy show modest success. In spite of this, several groups reported exciting results with therapeutic regimens that combine PPAR**γ** ligands with other compounds: chemotherapeutic agents, retinoid x receptor (RXR)**α** agonists, statins, or cell-to-cell signaling molecules in preclinical cancer models and human trials. Here we have compiled an extensive review, consolidating the existing literature, which overwhelmingly supports a beneficial effect of treating with PPAR**γ** ligands in combination with existing chemotherapies versus their monotherapy in cancer. There are many examples in which combination therapy resulted in synergistic/additive effects on apoptosis, differentiation, and the ability to reduce cell growth and tumour burden. There are also studies that indicate that PPAR**γ** ligand pretreatment overcomes resistance and reduces toxicities. Several mechanisms are explored to explain these protective effects. This paper highlights each of these studies that, collectively, make a very strong case for the use of PPAR**γ** ligands in combination with other agents in the treatment and management of several cancers.

## 1. Introduction

Cancer is the leading cause of death worldwide, with the projected number of associated deaths continuing to rise to an estimated 13.1 million people by 2030 [1]. For any given tumour, a concerted evaluation of type, stage, location, and size at the time of diagnosis influence the selection of one or more available treatment interventions, including surgery, radiotherapy, chemotherapy, or combinations as appropriate. Accordingly, improved understanding of how chemotherapeutic interventions can be optimized will assist with cancer prevention, as well as treatment and care of cancer patients.

Though many single agent treatments of solid or hematologic tumours are effective, they often select for resistant cells, and ultimately recurrent tumours, which no longer respond to the initial therapy [2]. To minimize the development of resistance, researchers and clinicians have expanded the use of combination drug therapies for some time. This approach favours combining individual classic chemotherapeutic agents aimed at forming new optimized regimens with additive/synergistic protective effects [3–5]. Of course, these combinations must also be chosen wisely to avoid similar synergism in toxicity. To achieve maximal chemotherapeutic potential and satisfy the previous conditions, many groups have explored combinations of traditional chemotherapies with the growing arsenal of targeted pathway-specific drugs [6], including those that activate an emerging target* peroxisome proliferator-activated receptor (PPAR)*γ**. This paper is a review of the vast *in vitro, in vivo,* and human clinical trial studies, irrespective of cancer type, using chemotherapeutic combinations that include PPAR*γ*-activating drugs. The aims are to evaluate the novel chemotherapeutic potential of PPAR*γ*-activating drugs and provide a guide for further basic and clinical research, in order to optimize chemotherapeutic interventions that will reduce the number of cancer-related deaths worldwide.

PPAR*γ* is a candidate tumour suppressor gene and member of the nuclear receptor superfamily [7]. The gene encodes two isoforms, PPAR*γ*1 and PPAR*γ*2, derived from alternative splicing, which are preferentially expressed in nonadipogenic cell types and cells committed to the adipocyte lineage, respectively [8–10]. PPAR*γ* normally associates with the retinoid X receptor (RXR)*α* and the resulting PPAR*γ*; RXR*α* complex recognizes direct-repeat- (DR-) 1 motifs, referred to as peroxisome proliferator response elements (PPREs), in the promoters of target genes [11]. Complexed PPAR*γ* is activated by ligands which include synthetic thiazolidinediones such as the gold standard activator rosiglitazone (ROSI) [12], used widely for >10 years to treat and prevent type II diabetes [13], as well as pioglitazone (PIO), troglitazone (TRO), ciglitazone (CIG), and many natural fatty acids and fatty acid metabolites, such as linoleic acid and signaling molecules like 15-deoxy-D^12,14^-prostaglandin J_2 _(15d-PGJ_2_) [14].

PPAR*γ* ligands are reported to exert antitumourigenic properties *in vitro* and to induce tumour growth arrest or shrinkage in murine *in vivo *models [15–19]. Based on this, a few clinical trials have been performed to evaluate the effectiveness of PPAR*γ* ligands in human cancer. In the most successful of these trials, three patients with advanced unresectable myxoid and pleiomorphic liposarcoma were treated with TRO. Serial biopsies revealed increased lipid accumulation, indicative of adipocyte differentiation, and a 2- to 4-fold decrease in the percentage of cells expressing the Ki-67 antigen, a marker of proliferation [20]. Unfortunately, further monotherapy trials using PPAR*γ* ligands on more common epithelial-based cancers have not been as fortuitous. In separate phase II clinical trials, 22 women with refractory breast cancer and 25 patients with advanced colorectal cancer, respectively, treated with TRO experienced no objective tumour responses [21, 22]. Similarly, ROSI treatment did not prolong time to disease progression compared to placebo in 106 men with prostate carcinoma [23] or affect proliferation in breast tumours during a short pilot study [24].

Despite the limited success as a monotherapy, PPAR*γ* agonists have shown tremendous potential for clinical utility when combined with traditional chemotherapeutics, RXR*α* ligands, statins, and cellular signaling molecules. Substantial evidence suggests that activating PPAR*γ* synergistically enhances the protective effects of these agents, reduces their inherent toxicity, and even, in some cases, overcomes resistance. A summary of the preclinical and clinical work combining PPAR*γ* ligands with various other compounds is provided in Tables 1 and 2, respectively. Extensive literature searches were performed using the US Library of Medicine and National Institute of Health's http://www.ncbi.nlm.nih.gov/pubmed/ for papers using treatment regimens that combined PPAR*γ* agonists with other therapeutic agents. Any errors by omission are unintentional.

## 2. Chemotherapeutic Agents

### 2.1. Platinum Compounds

Platinum-based compounds have been widely used as chemotherapeutics since the 1970s to treat cancers of the breast, lung, ovary, testis, head, and neck [25]. These agents exert their cytotoxic effects by cross-linking DNA, which impairs DNA transcription and replication [26]. This damages cells which invoke DNA repair mechanisms and, when those fail, apoptosis [27]. Cisplatin, the first such compound available, is an extremely effective chemotherapeutic, although dosing is limited due to the associated risk of nephrotoxicity [28, 29]. Second and third generation drugs, carboplatin and oxaliplatin, are less damaging to kidneys but are associated with severe neuropathies [30]. PPAR*γ* ligands in combination with platinum-based compounds have increased therapeutic efficacy, overcome resistance, and decreased toxicity in multiple cancer models.

Several cancer cell lines, including A549, Calu1, H23, H596, and H1650 non-small-cell lung cancer (NSCLC), Mosher colon cancer, and OVCA420, OVCA429, and ES2 ovarian cancer cells have demonstrated the synergy of combination treatment with platinum-based compounds and therapeutic doses of ROSI. These cells exhibited greater growth reduction, G2-M arrest, and increased apoptosis when treated with the combination than either agent, ROSI or chemotherapeutic, alone. *In vivo* xenograft mouse models using A549 lung cancer cells also suggest synergy, as low doses of ROSI and carboplatin reduced xenografted tumours to one-third the size of tumours from monotherapy controls [31]. In a separate study, ROSI pretreatment resulted in maximum reduction in mammary tumour volume when combined with cisplatin compared to treatment with cisplatin alone. The mammary tumours from cotreated mice also exhibited more glandular structures suggesting improved differentiation, an indication of less aggressive tumours which, clinically, would have a better prognosis [32]. Interestingly, another study, using TRO in combination with cisplatin in A549 and H522 non-small-cell lung cancer cells, found synergistic effects when TRO treatment followed cisplatin treatment but not vice versa, suggesting that the beneficial effects of PPAR*γ* activation might depend on the sequence of drug administration [33]. The combinational regimen may also be effective to treat malignant pleural mesothelioma as TRO and cisplatin have an additive effect on EHMES-10 cells *in vitro* as well as tumour growth reduction and overall survival in xenograft mouse models, compared to either agent singularly in an animal model [34].

Many tumours, including ovarian and non-small-cell lung, that are initially responsive to platinum-based compounds eventually develop resistance [35]. The accruing resistant tumours grow unabated and are associated with poor prognosis [36]. Resistant tumours use multiple survival strategies including altered drug-uptake pathways, which prevent platinums from reaching DNA, or decreased DNA damage recognition and apoptosis network signaling [26]. 

Interestingly, combination treatment with PPAR*γ* activators may be able to overcome this resistance. In one study, mice with EGFR- and K-Ras-driven lung adenocarcinomas, a model of platinum-resistant lung cancer, were treated with carboplatin, ROSI, or both. Neither monotherapy reduced tumour burden; however, combination therapy resulted in 80% reduction in tumour volume [57]. Microarray analysis from a separate study revealed that ROSI treatment reduces expression of five members of the metallothionein gene family [31]: metal-binding proteins that play a crucial role in platinum-drug resistance by sequestering platinum compounds outside the cell [61].

In addition to developed resistance, platinum-based compounds are associated with several morbidities, including nephrotoxicity, myelosuppression, and GI complications [26]. Given this, and the potential for an additional drug, in this case a PPAR*γ* ligand, to exacerbate the inherent toxicity of platinums, the authors of the aforementioned lung adenocarcinoma study conducted extensive toxicological analysis on their treated mice. Fortunately, compared to monotherapy, combination therapy did not decrease markers of immune function, white blood cell counts, or hematocrit, and BUN and creatinine levels, indicative of kidney damage, were similarly unaffected [57].

Nephrotoxicity, experienced by 28–36% of patients after a single injection of cisplatin [62], may be, in part, exacerbated by TNF-*α*, a well-known mediator of inflammation [40]. Interestingly, PPAR*γ* activators reduce inflammatory responses [63, 64]. Therefore, Tikoo et al. used a DMBA-induced murine breast cancer model to evaluate the ability of ROSI to decrease nephrotoxicity. They found that ROSI pretreatment significantly decreased circulating BUN, creatinine and TNF-*α*, and minimized tubular damage, suggesting that PPAR*γ* activation ameliorated the nephrotoxicity associated with cisplatin treatment [32]. If this holds true in humans, ROSI treatment may allow physicians to use platinum-based compounds at higher, previously toxic, doses that may confer additional therapeutic benefit.

### 2.2. Taxanes

Taxanes, including paclitaxel and docetaxel, are commonly used chemotherapy agents for a large array of cancers which include ovarian, lung, head and neck, esophageal, breast, prostrate, and gastric cancers. Taxanes exert their effects by binding and immobilizing microtubules which prevents cell division [65]. There are multiple side effects associated with taxanes including reduced hematocrit, neuropathy, and myalgias/arthralgias [66].

A novel high-affinity PPAR*γ* agonist, and thiazolidinedione derivative, RS5444, demonstrated additive antiproliferative activity on DRO90-1 and ARO81 anaplastic thyroid carcinoma cells, a particularly aggressive and dedifferentiated cancer [67]. RS5444 did not induce apoptosis by itself; however, when combined with paclitaxel, the apoptotic fraction of cells doubled. Using IC_25_ values experimentally derived from *in vitro* experiments, the group found that combination treatment with RS5444 and paclitaxel significantly reduced xenograft tumour volumes compared to either monotherapy alone [53].

Non-small-cell lung cancer is a leading cause of death from malignant disease in industrialized nations with a 5-year survival rate of approximately 15% [68, 69]. Novel therapeutic regimens involving PPAR*γ* activators and traditional chemotherapeutics have shown some promise that they may someday improve this rate. An *in vitro* study indicated synergy between multiple PPAR*γ* ligands (TRO and PIO) and paclitaxel in A549, H522 non-small-cell lung cancer cells that was dependent upon treatment order, with paclitaxel preceding TRO treatment [33]. Another group confirmed the synergistic effect of combining PPAR*γ* activation with, this time, docetaxel. In this study, 15d-PJ_2_ increased cytotoxicity in A549 and H460 cells *in vitro. *Extending this, they found that 15d-PJ_2_ and docetaxel reduced A549 and H460 xenografted tumour volumes by 72%, nearly double the effect of docetaxel alone [54].

### 2.3. Topoisomerase Inhibitors

Both classes of topoisomerase inhibitors, type I (including irinotecan) and type II, work by binding and incapacitating topoisomerases: enzymes that are critical for DNA supercoiling and strain relief [70]. Ultimately, this binding prevents movement of the DNA replication fork which induces stress responses that can lead to apoptosis or the involvement of DNA damage repair mechanisms [71]. A topoisomerase I inhibitor, irinotecan, has demonstrated activity against a vast range of cancers [72] but is associated with significant GI toxicity and myelosuppression [73]. Budman and Calabro have shown synergistic cytotoxic increases in a variety of cell lines (MCF-7, MCF-7/adr, and SK-BR-3 breast cancer; H460 lung cancer; SW480 and RT4 colon cancer; HT1197 bladder cancer) between irinotecan and the PPAR*γ* ligand LY293111 at clinically attainable doses [6], prompting human studies with this drug combination. To date, a phase I clinical trial has established a dosing schedule that minimized adverse GI events associated with LY293111 and irinotecan [59]. Another topoisomerase I inhibitor, camptothecin, enhanced the cytotoxicity of 15d-PGJ_2_ in Cak-2 renal cell carcinoma cells. Interestingly, the authors did not find synergy when 15d-PGJ_2_ was combined with other chemotherapeutics including doxorubicin, 5-FU, and cisplatin [55]. This synergism may allow clinicians to reduce the dose of topoisomerase inhibiting agents and thereby reduce associated toxicity, by combining treatment with PPAR*γ* ligands.

### 2.4. Antimetabolites

Antimetabolites, including 5-Fluorouracil (5-FU), methotrexate, and others, are structurally similar compounds to vitamins, amino acids, or nucleic acid precursors which become incorporated into cellular macromolecules with disastrous consequences for cells such as inhibition of cell growth and division [74]. They have been used to treat several types of cancer including leukemia, breast, and ovarian but have been associated with myelosuppression, dermatitis, and diarrhea [75]. A phase II clinical trial was undertaken to evaluate the role of capecitabine, a precursor to 5-FU, in combination with PIO to treat recurrent high-grade gliomas. Only 29% of patients experienced disease stabilization after three months; however, the regimen was well tolerated by patients indicating potential for future therapeutic utility [58].

Hepatocellular carcinoma (HCC) and colorectal tumours are among the leading forms of cancer contributing to cancer-related deaths [69, 76]. HCC usually requires chemotherapy because tumours are often surgically unresectable due to advanced stage at diagnosis [77]. Treatment of both diseases often involves 5-FU; however, patients often respond poorly as tumours develop multiple drug resistance [78–80] due to multiple mechanisms including increased drug efflux [81]. Interestingly, PPAR*γ* may regulate ABC transporters, key proteins involved in drug efflux [82]. Accordingly, activation of PPAR*γ* with ROSI, in combination with 5-FU treatment, has been evaluated in HCC and colon cancer. ROSI treatment decreased cell viability in two HCC cell lines (BEL-7402 and Huh-7) by 4- and 2-fold, respectively, compared to treatment with 5-FU alone. The authors also used siRNA to show that this effect was dependent on PPAR*γ* [37]. Another group evaluated ROSI treatment with 5-FU in HT-29 colon cancer cells and found that ROSI treatment, at a low dose that did not affect proliferation or cell growth, enhanced 5-FU-induced apoptosis. Again, this effect was PPAR*γ* dependent as it was ameliorated by the PPAR*γ* antagonist GW9662 [83].

Another antimetabolite, gemcitabine, is a useful chemotherapeutic that arrests cell growth in multiple ways including incorporation into DNA and impeding cell division [84]. Gemcitabine is standard therapy for pancreatic cancer, a disease with a strikingly poor prognosis as most patients die within six months of diagnosis [85]. Gemcitabine only modestly prolongs survival but is useful as a palliative agent for several cancer-related morbidities. Hennig et al. evaluated the ability of the PPAR*γ* activator LY293111 to enhance the activity of gemcitabine in an orthotopic pancreatic cancer model. Consistent with previous models, both gemcitabine and LY293111 significantly inhibited tumour growth and reduced the incidence of liver metastasis; however, the combination was more effective than either therapy alone. Furthermore, combination treatment maintained stable body weights, relieved tumour-induced cachexia, and decreased incidence of bowel obstruction [60]. This suggests that this combination may be effective, to not only treat aggressive pancreatic adenocarcinomas but also relieve side effects associated with monotherapy [86].

### 2.5. RXR*α* Ligands

The PPAR*γ* binding partner, RXR*α*, is also a member of the nuclear receptor superfamily. RXR has three subtypes (**α**, **β**, and **γ**), which are activated by retinoids, a group of vitamin A analogues. After ligand binding, RXR*α* is able to modulate gene expression by binding retinoid X receptor responsive elements (RXREs), present in the promoter regions of target genes. Similar to PPAR*γ*, RXR*α* activation profoundly affects multiple cellular activities that are pertinent to cancer including cellular growth, differentiation, apoptosis, and morphogenesis [87, 88].

Given this, multiple groups have investigated the combined use of PPAR*γ* and RXR*α* ligands. The first report, from Tontonoz et al., indicated that simultaneous treatment of liposarcoma cells, selected from freshly harvested tumours, with both RXR*α*- and PPAR*γ*-specific ligands, synergistically stimulated differentiation. Additionally, the authors showed that PPAR*γ* is highly expressed in the major histological types of liposarcoma, suggesting that PPAR*γ*-targeting agents, especially combined with RXR*α* ligands, may be useful therapy for human liposarcoma [45].

Since that time, beneficial effects have been reported for several types of malignancies, including hematological, breast, and lung cancer, for the combined treatment of PPAR*γ* ligands and retinoids. Konopleva et al. reported that PPAR*γ* is expressed in lymphoid (Su-DHL, Sup-M2, Ramos, Raji, Hodgkin's cell lines, and primary chronic lymphocytic leukemia) and myeloid (U937 and HL-60) cell lines, several of which undergo apoptosis when treated with PPAR*γ* ligands including ROSI and 15d-PGJ_2_. The apoptotic effects of PPAR*γ* ligands were enhanced when combined with an RXR*α* agonist, LG100268, as reflected by mitochondrial depolarization and caspase activation [38]. Similarly, Ray et al. showed that PPAR*γ* is expressed in ANBL6 and 8226 human multiple myeloma cell lines and that PPAR*γ* ligands induce apoptosis, an effect which is enhanced by combination with 9-cis retinoic acid, a ligand of RXR*α* [51]. Elstner et al. found that PPAR*γ* ligands were potentiated by RXR*α* ligands in multiple breast cancer cells (MCF-7, T-47D, ZR-75-) [42], work that was later confirmed by multiple groups [41, 56] including one study that also found protective effects in Calu-6 lung cancer cells [48]. One of these studies showed that combination treatment with ROSI and the RXR*α* ligand 9-cis retinoic acid inhibited cell viability in MCF-7, MCF-7TR1, SKBR-3, and T-47D breast cancer cells but did not affect MCF-10a normal immortalized breast epithelial cells. This exciting work suggests that the cytotoxic effect maybe specific to cancer cells. Mehta et al. took this approach into mouse models and found that the combination of LG10068, an RXR*α*-specific ligand, and TRO completely inhibited development of mammary tumours at micromolar concentrations in a DMBA-induced breast tumourigenesis model [17].

Many potential mechanisms are postulated to explain the synergistic protective effects of PPAR*γ* and RXR*α* ligands. The protective effect at the whole body level may, in part, be mediated at the transcriptional level by the ability of PPAR**γ**and RXR*α* ligands to inhibit aromatase secretion [89], enhance expression of glutathione S-transferases (GSTs) [90], or downregulate expression of matrix metalloproteinases (MMPs) [52]. Aromatase catalyzes the rate-limiting step in estrogen biosynthesis [91], which drives breast tumourigenesis by stimulating proliferation of breast tumour cells [92]. GSTs have multiple functions including the detoxification of several xenobiotics and carcinogens [93]. MMPs degrade extracellular matrix proteins carving pathways for migrating cancer cells and releasing sequestered growth factors [94]. Combined RXR*α* ligand and CIG treatment decreases cell growth and the invasive potential in G361 melanoma and U87MG glioblastoma cells by decreasing expression of matrix metalloproteinases [52].

Other groups have theorized that the synergistic effects of PPAR**γ**and RXR*α* ligands may not be directly related to transcriptional effects. Ligands of PPAR**γ**and RXR*α* recruit different subsets of transcriptional coactivators [95]; therefore, cotreatment may increase transcription as there is less competition [96]. Additionally, PPAR*γ* activity may enhance proteosome inhibitors, allowing for RXR*α* accumulation and the enhancement of PPAR*γ* : RXR*α*-mediated transcription [97]. Collectively, this work suggests that combining agents that activate both PPAR*γ* and RXR*α* could synergistically enhance the protective effects of either agent alone.

## 3. Cell Signaling Molecules

Protective synergy with PPAR*γ* ligands is not exclusive to traditional chemotherapeutic agents or RXR*α* ligands. There are a few reports of PPAR*γ* activators combining with normal cell signaling molecules, including tumour necrosis factor (TNF)*α*, tumour necrosis factor-related apoptosis-inducing ligand (TRAIL), and Heregulin to confer an additive or synergistic protective effect. TNF*α* is a cytokine, chiefly produced by activated macrophages, that is involved in systemic inflammation, and leads to tumour regression [98, 99]. TRAIL, a member of the TNF family, induces apoptosis by binding receptors and recruiting the Fas-associated death domain and caspase-8, triggering apoptosis [100]. Heregulin is a soluble secreted growth factor that activates several classic tumourigenic signal transduction pathways including PI3K/Akt, Ras/MAPK, and JNK [101].

Based on evidence that ROSI upregulates p53 and p21, Mody et al. examined the ability of ROSI pretreatment to sensitize MDA-MB-231 breast cancer cells to therapies that act on these apoptosis/cell death pathways, such as TNF*α*. ROSI pretreatment dramatically increased TNF*α*-mediated growth inhibition by 9-fold versus control TNF*α* or ROSI alone. The authors also performed microarray analysis to evaluate genetic changes associated with ROSI treatment [39]. This may be a valuable tool to predict other agents which synergize with PPAR*γ* ligand activity based on shared pathway utilization.

Partridge and Barnes evaluated the ability of multiple PPAR*γ* ligands (CIG, TRO, and 15d-PGJ_2_) to enhance the efficacy of TRAIL in a drug-resistant ovarian cancer cell line. Drug resistance is a serious problem in ovarian cancer, especially in advanced disease, where survival rates fall to 10–30% [102]. The combined treatment with CIG and TRAIL synergistically reduced proliferation in multiple cell lines, most notably the paclitaxel-resistant HEY ovarian cancer subclone. TRO treatment showed no effect on proliferation on its own; however, when combined with TRAIL, that reduced cell numbers in etoposide-, pemetrexed-, cisplatin-, docetaxel-, and gemcitabine-resistant cell lines. Similarly, 15d-PGJ_2_ treatment inhibited growth in all cell lines, especially the HEY cell line which was developed by the authors [43].

Park et al. showed that Heregulin, which paradoxically drives tumourigenesis [103, 104], synergistically increases TRO-mediated breast cancer cell apoptosis and necrosis *in vitro* [44]. In light of previous reports that Heregulin plays a causal role in Tamoxifen- and Gefitinib-resistant breast cancer [105], Park's work provides evidence that combination therapy with Heregulin and PPAR*γ*-activators may be a novel strategy for the treatment of resistant or refractory breast cancer [44].

## 4. Statins

Statins are another important class of drugs acting as inhibitors of 3-hydroxy-3-methylglutarylcoenzyme A (HMG-CoA) reductase, a critical rate-limiting enzyme in cholesterol biosynthesis. Statins are commonly used to manage hypercholesterolemia and cardiovascular diseases and are some of the most frequently prescribed therapeutics for elderly patients. Recently, statins were evaluated for their protective effects in cancer and showed antiproliferative and pro-apoptotic effects *in vitro* [106–108]. Incubation with lovastatin and CIG for 48 hrs exerted additive cytotoxic and cytostatic effects in multiple cancer cell lines (Panc 02 and MIA PaCa-2 pancreatic cancer, C-26 colon cancer, and EMT6 and MDA-MB-361 breast cancer) compared to either treatment alone [49, 50]. Further experiments on human U87, U138, LN 405, and rat RG II glioblastoma cells indicated cytotoxic synergy after 48- and 144-hour treatments with PIO and a variety of statins [46]. Additionally, treatment of two meningioma cell lines (IOMM-Lee and KT21-MG1) with PIO and statins showed significant synergistic cytotoxic effects [47]. It was also suggested that statins may signal through the transcription factor sterol response element-binding protein (SREBP) to encourage PPAR*γ*-mediated upregulation of PTEN [109]. This evidence suggests yet another class of drugs that, combined with PPAR*γ*-ligands, show synergistic protective effects in cancer.

## 5. Areas Needing More Work

The majority of literature in the field supports the view that combination cancer therapy with PPAR*γ* ligands and chemotherapeutic agents produce beneficial effects. However, this trend is not universal. Multiple groups have evaluated combinations of chemotherapeutic cocktails that include PPAR*γ* ligands and found no synergism. For example, Yamamoto et al. reported that the synergistic toxic effects of 15d-PGJ_2_ in renal cell carcinoma were specific to its combination therapy with camptothecin, and not evident with two common chemotherapeutic agents: 5-fluorouracin and cisplatin [55]. Tapia-Perez's group also found that synergism in glioblastoma cells depends on the combination, this time the PPAR*γ* ligand, as PIO + statin treatment produced a significant cytotoxic effect although the same was not true for ROSI + statin [46] Clearly, more work is needed to establish which combinations will be effective in which diseases, work that will be further complicated by factors such as dose and treatment timing (pre-, post-, cotreatment, etc.). Interestingly, the same combination regimen may not always be effective, even within the same disease, as Elstner et al. reported that only three (MCF-7, MDA-MB-231, and ZR-75-1) of the eight (MCF-7, BT20, BT474, MDA-MB-231, MDA-MB-436, SKBR3, T-47D, ZR-75-1) breast cancer cell lines they evaluated were sensitive to combinations of the PPAR*γ* ligand TRO and RXR*α* ligand 9-cis retinoic acid. Interestingly, the sensitive cell lines all express high levels of the apoptosis protein bcl-2 [42]. This underscores the importance of work to evaluate the molecular mechanisms by which combination therapies exert their effects so that, someday, clinicians and researchers may predict treatment efficacy using molecular signatures. Most notably, extensive literature searches did not reveal reports of PPAR*γ* ligands impeding the therapeutic efficacy of chemotherapeutic agents.

Synthetic PPAR*γ* ligands are generally well-tolerated and nontoxic; however, multiple groups have reported adverse cardiovascular events associated with PPAR*γ* ligands, including myocardial hypertrophy and congestive heart failure due to plasma volume expansion and edema, in humans and animal models [110–112]. To address this problem, the FDA convened leading experts in 2010 to carry out more research to definitively show whether PPAR*γ* ligands are associated with increased cardiovascular risk. The committee observed no significant difference in acute myocardial infarction and acute heart failure between patients treated with ROSI or PIO versus matched control cases and recommended that further studies be performed to address this issue [113, 114]. While these studies are in progress, research should continue to evaluate PPAR*γ* ligands for their efficacy and mechanisms of action given their well-documented protective effects in many diseases, including, but not limited to, cancer. Furthermore, a better understanding of the mechanisms by which activation of PPAR*γ*-dependent signaling stops tumourigenesis may provide the basis for future development of more efficacious drugs to prevent and/or reduce cancer-related deaths.

## 6. Discussion

The studies summarized previously, and in Tables 1 and 2, suggest that the combination of PPAR**γ**ligands plus standard chemotherapeutic agents, RXR*α* agonists, statins, and certain cellular signaling molecules holds promise as a novel therapy for several types of malignancy. In general, combined use of two or more therapeutic compounds is often advantageous because of the potential to use lower clinical doses of each, which decreases nonspecific toxicity. However, here we report several examples of synergistic/additive interactions between agents that activate PPAR**γ** as well as reductions in toxicity and the ability to overcome resistance. The results here are largely preclinical, with a select few regimens being evaluated in human subjects and, even in those cases, early clinical trials which, naturally, focused largely on toxicity as opposed to efficacy. However, the volume of preclinical evidence suggests that a breakthrough in the clinical application of combination therapy with PPAR*γ* agonists is very possible. Moving forward, studies should continue to evaluate mechanisms by which these regimens induce their therapeutic effects as this will ultimately lead to identification of patient populations with high probabilities of therapeutic efficacy. In summary, the types of combination therapy described here are promising strategies for the chemoprevention, management, and/or treatment of several types of cancer.

## Figures and Tables

**Table 1:** Synergistic effects between PPAR*γ* ligands and other agents *in vitro*. Descriptions reflect most noteworthy finding of each study.

	Platinum-Based Compounds	Taxanes	Topoisomerase Inhibitors	Anti-metabolites	RXR*α* ligands	Cell Signalling Molecules	Statins
Rosiglitazone	(i) ↓ Cell growth in A549, Calu1, H23, H596 and H1650 NSCLC; Mosher colon; and OVCA420, OVCA429 and ES2 ovarian cancer [31]			(i) ↓ Cell viability in BEL-7402 and Huh-7 Hepatocellular carcinoma [37]	(i) ↑ Differentiating and growth-inhibitory effects in lymphoid Su-DHL, Sup-M2, Ramos, Raji, Hodgkin's cell lines, and primary chronic lymphocytic (lymphoid), and U937 and HL-60 (myeloid) leukemia [38]	(i) ↑ Growth inhibition in MDA- MB-231 breast cancer [39] *pre-treatment	
				(ii) ↑ Apoptosis in HT-29 colon cancer [40]	(ii) ↓ Cell viability in MCF-7, MCF-7TR1, SKBR-3, and T-47D breast cancer [41]		

Troglitazone	(i) ↑ Growth inhibition in A549 and H522 NSCLC [33] *post-treatment	(i) ↑ Growth inhibition in A549 and H522 NSCLC [33] *post-treatment			(i) ↑ Growth inhibition in MCF-7, T-47D, ZR-75-1 breast cancer [42]	(i) ↓ Cell numbers in HEY ovarian cancer [43]	
	(ii) ↑ Growth inhibition in EHMES-10 mesothelioma [34]					(ii) ↑ Apoptosis in MCF-7, SKBR-3, and MDA-MB-453 breast cancer [44]	

Pioglitazone		(i) ↑ Growth inhibition in A549 and H522 NSCLC [33] *post-treatment			(i) ↑ Differentiation in harvested liposarcoma cells [45]		(i) Cytotoxic effects in U87, U138, LN 405 and rat RG II glioblastoma [46]
							(ii) ↑ Cytotoxic effects in IOMM Lee and KT21-MG1 meningioma [47]

Ciglitazone					(i) ↑ Growth inhibition in ZR-75-1 and T-47D breast; and Calu-6 lung cancer [48]	(i) ↓ Proliferation in HEY ovarian cancer [43]	(i) ↑ Cytotoxic and cytostatic effects in Panc 02 and MIA PaCa-2 pancreatic; C-26 colon; and EMT6 and MDA-MB-361 breast cancer [49]
					(ii) Apoptosis in ANBL6 and 8226 multiple myeloma [50]; C6, U87MG andGL261 glioblastoma [51],and G361 melanoma [50, 52]		

RS5444		(i) ↑ Antiproliferative effects in DRO90-1 and ARO81 anaplastic thyroid carcinoma [53]					

15d-PGJ2		(i) ↑ Cytotoxicity in A549 and H460 NSCLC [54]	(i) ↑ Cytotoxicity in Cak-2 renal cell carcinoma [55]		(i) ↑ Differentiating and growth-inhibitory effects in Su-DHL, Sup-M2, Ramos, Raji, Hodgkin's cell lines, and primary chronic lymphocytic (lymphoid), and U937 and HL-60 (myeloid) leukemia [38]	(i) ↑ Growth inhibition in HEY ovarian cancer [43]	
					(ii) ↑ Apoptosis in ANBL6 and 8226 multiple myeloma [51]		

LY 293111			(i) ↑ Cytotoxicity in MCF-7, MCF-7/adr and SKBR-3 breast; H460 lung; SW480 and RT4 colon; and HT1197 bladder cancer [6]				

Linoleic Acid					(i) ↑ Apoptosis and ↓ proliferation in MCF-7, MDA-MB-231, MDA-MB-468, T-47D and SKBR-3 breast cancer [56]		

**Table 2:** *In vivo *and clinical trials synergistic effects between PPAR*γ* ligands and other agents. Descriptions reflect most noteworthy finding of each study.

	Platinum-based compounds	Taxanes	Topoisomerase inhibitors	Anti-metabolites
Rosiglitazone	(i) ↓ Volume of A549 NSCLC xenografted tumours [31]			
	(ii) ↓ Volume in KRAS- and EGFRdriven lung tumours without disrupting immune system [57]			
	(iii) ↓ Volume and ↑ differentiation of DMBA-induced breast tumours; treatment minimized nephrotoxicity [32] *pre-treatment			

Troglitazone	(i) ↓ Volume and ↑ overall survival of EHMES-10 malignant pleural mesothelioma xenografted tumours [34]			

Pioglitazone				*Phase 2 clinical trial: * (i) 30% of patients with high grade gliomas experienced disease stabilization; treatment well tolerated by all [58]

RS5444		(i) ↓ Volume of DRO90-1 and ARO81 anaplastic thyroid carcinoma xenografted tumours [53]		

15d-PGJ_2_		(i) ↓ Volume of A549 and H460 NSCLC xenografted tumours [54]		

LY 293111			*Phase 1 clinical trial*: (i) ↓ GI toxicity in patients with advanced solid tumours [59]	(i) ↓ Volume of S2-013 pancreatic xenografted tumours; minimized side effects [60]
